# The Kinocilia of Cochlear Hair Cells: Structures, Functions, and Diseases

**DOI:** 10.3389/fcell.2021.715037

**Published:** 2021-08-05

**Authors:** Difei Wang, Jun Zhou

**Affiliations:** ^1^State Key Laboratory of Medicinal Chemical Biology, College of Life Sciences, Nankai University, Tianjin, China; ^2^Shandong Provincial Key Laboratory of Animal Resistance Biology, Collaborative Innovation Center of Cell Biology in Universities of Shandong, Institute of Biomedical Sciences, College of Life Sciences, Shandong Normal University, Jinan, China

**Keywords:** primary cilia, ciliopathy, auditory system, hair cell, kinocilia

## Abstract

Primary cilia are evolutionarily conserved and highly specialized organelles that protrude from cell membranes. Mutations in genes encoding ciliary proteins can cause structural and functional ciliary defects and consequently multiple diseases, collectively termed ciliopathies. The mammalian auditory system is responsible for perceiving external sound stimuli that are ultimately processed in the brain through a series of physical and biochemical reactions. Here we review the structure and function of the specialized primary cilia of hair cells, termed kinocilia, found in the mammalian auditory system. We also discuss areas that might prove amenable for therapeutic management of auditory ciliopathies.

## Introduction

Primary cilia are non-motile, highly specialized, and evolutionarily well-conserved organelles that project from the cell surface, which are essential throughout biological development and maturation. As analytical technologies have developed over the last few decades, our understanding of primary cilia has gradually changed from regarding them neglected “degenerate organelles” to well-appreciated “cellular antennas” (Singla and Reiter, 2006). There is only one primary cilium per cell (Satir and Christensen, 2007). The ciliary membrane of primary cilia harbors a variety of receptors and ion channels, including components of the Notch, Hedgehog, Wnt, G protein-coupled receptor, receptor tyrosine kinase, transforming growth factor β/bone morphogenetic protein signaling pathways, and Ca^2+^ channel-associated proteins such as polycystin 1 and 2 (Delling et al., 2013; Christensen et al., 2017; Anvarian et al., 2019; Ta et al., 2020). Through these cilium-dependent signaling pathways, primary cilia play key roles in the regulation of cell division, proliferation, and signal transduction and are thus crucial in tissue and organ development and normal mammalian physiology (Lancaster and Gleeson, 2009; Goetz and Anderson, 2010; Joukov and De Nicolo, 2019; Nachury and Mick, 2019). Moreover, primary cilia can act as a portal connecting the organism to the environment (Falk et al., 2015; Bujakowska et al., 2017; Uytingco et al., 2019; Ran and Zhou, 2020).

Some primary cilia with specialized structures and functions have been characterized in sensory cells, which can transduce external physical or chemical signals, such as smell and visual signals, to electrical signals in mammalian olfactory and vision systems (Falk et al., 2015). Kinocilia are specialized primary cilia present in auditory hair cells (HCs) in the inner ear. These cilia do not directly mediate auditory mechano-electrical transduction (MET), but partially retain the characteristics of motility responsible for the response of HCs to sound stimuli. Although showing a traditional 9 + 2 axoneme pattern of “motile cilia,” they lack the inner dynein arms and only directionally “move” after the cells sense sound, i.e., passive motion (Kikuchi et al., 1989). Besides, kinocilia are essential for HCs morphogenesis and planar cell polarity (PCP), and further auditory integrity (Sipe and Lu, 2011; Kazmierczak and Muller, 2012; Elliott et al., 2018). Genetic mutations affecting ciliary proteins can lead to diseases in multiple organs, collectively known as ciliopathies. Therefore, maintaining stable morphology and structure of kinocilia is essential to normal physiology and their dysfunction results in corresponding sensory ciliopathies. In this review, we describe the structure, function, and degeneration of kinocilia present in the mammalian auditory system and discuss if they are promising therapeutic targets for hearing deficits.

## Cochlear Hair Cells

The mammalian ear consists of the outer, middle, and inner ears, the latter consisting of the vestibular system and the cochlea. The former is sensitive to position signals mainly caused by linear acceleration and head rotation, and the latter mediates the conversion of vibrations into nerve impulses in response to sound (Liu et al., 2016). Both of these two systems have their own sensory epithelium, on which exist a large number of HCs that underpin both balance sensation and hearing. In the vestibular system, the sensory epithelium organizes as a repeating mosaic which consists of supporting cells and type I and type II HCs that differ in morphology and physiology (Warchol et al., 2019). However, the cochlea, a structure unique to mammals, has a more delicate sensory epithelium, also known as the organ of Corti.

**FIGURE 1:**
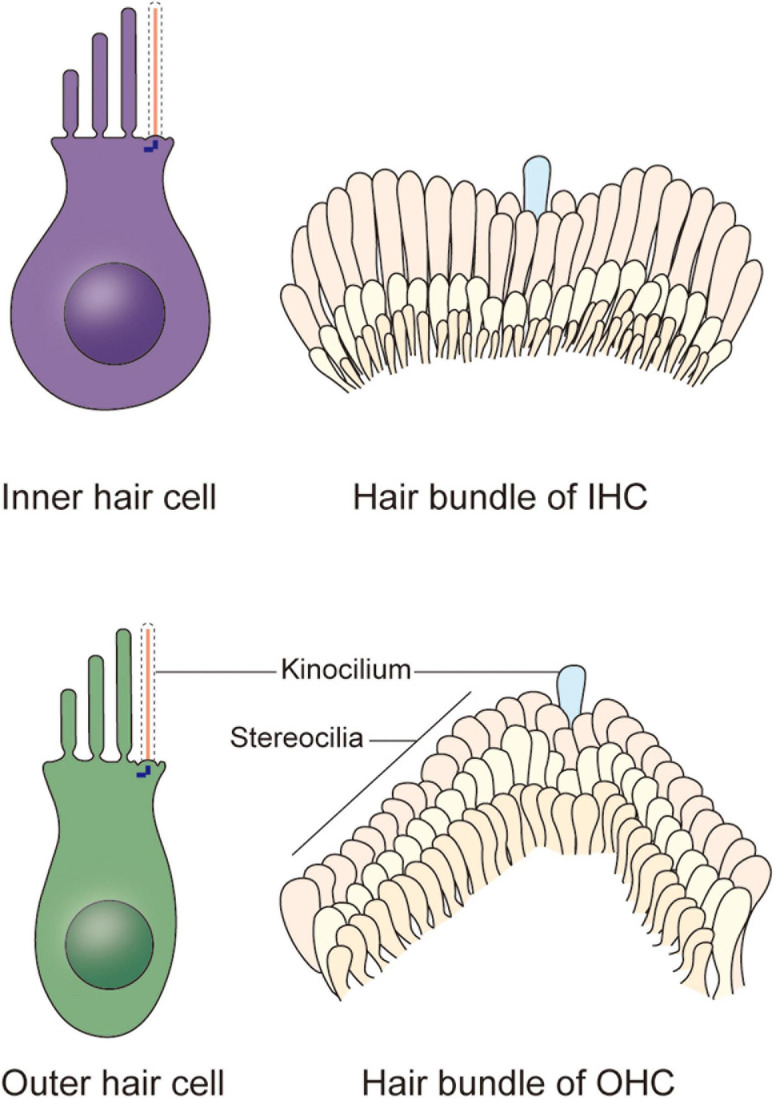
Schematic representation of the cochlear hair cells and hair bundles. Two types of hair cells responsible for mechano-electrical transduction and auditory sensing are present in the cochlea. The stair-like W or V-shaped hair bundle appears on the apical plasma membrane of each inner hair cell and outer hair cell, collectively. Each hair bundle contains plenty of stereocilia and a kinocilium near the corner of them, and the kinocilium degenerates after maturation of hair cells, indicating the acquisition of hearing.

In the organ of Corti, HCs are categorized as inner hair cells (IHCs) and outer hair cells (OHCs) (Figure 1; Atkinson et al., 2015). Every HC is supported by several highly specialized cells, such as Deiters’ cells, pillar cells, inner border cells, and Hensen’s cells. All of the HCs are highly differentiated and polarized, and each acts as a mechano-electrical transducer that turns physical signals into electrical signals. External sensory stimuli physically open MET channels leading to an influx of K^+^ ions that depolarizes the HC. HC depolarization activates Ca^2+^ channels at the plasma membrane resulting in neurotransmission onto spiral ganglion neurons via the cochlear nerve. Finally, physical signals turn into electrical signals, which then pass through spiral ganglion neurons via the cochlear nerve, and the sensory signal ultimately reaches the cortex via the auditory pathway (Fettiplace, 2017; Ashmore, 2020). The apical surface of HCs are arranged as a unique subset by a single row of IHCs and a triple row of OHCs, each of which is surrounded by a variety of non-sensory support cells based on their location relative to the spiral ganglion (Elliott et al., 2018; Tarchini and Lu, 2019). The OHCs are located on the lateral (non-neural) side and are mainly responsible for amplifying acoustic vibrations through periodic contraction and elongation of the cell body driven by changes in membrane potential. The IHCs are located on the medial (neural) side, where they integrate and transmit sound signals to neurons. Synergy between these two types of cells greatly improves the resolution and sensitivity of sound signal processing (Fettiplace, 2017).

## The Kinocilia of Cochlear Hair Cells

**FIGURE 2:**
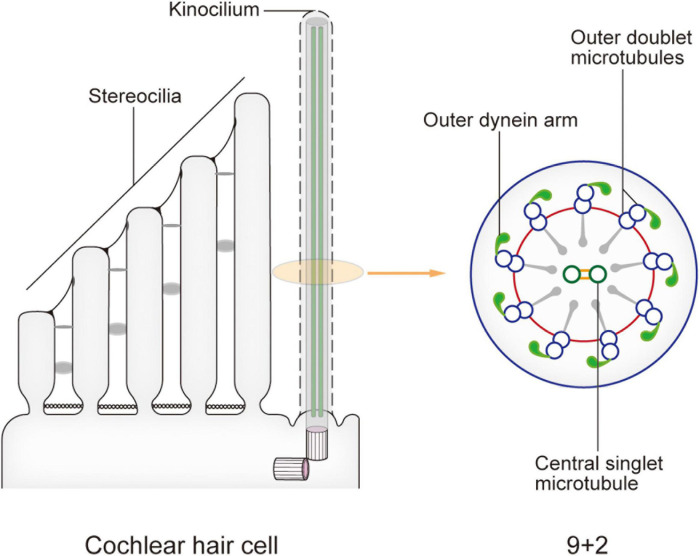
Model of the kinocilium and its cross section. The kinocilium shows a 9 + 2 axoneme pattern with typical motile cilium structures such as outer dynein arms and radial spokes. However, a lack of inner dynein arms renders the lack of motor function. After the kinocilium degenerates, stereocilia mediate the entire mechano-electrical transduction (MET) process. When sound waves are transmitted to the cochlea, the shearing motion caused by lymph flow drives the passive swing of the hair bundle and sound signal processing.

In newborn mice, the top of each HC possesses dozens to hundreds of actin filament-based stereocilia of increasing height arranged in a stepped V or W shape (Figure 1). A true microtubule-based cilium that is about the same height as the tallest row of stereocilia, called the kinocilium, is found near the corner of this arrangement, i.e., on the non-neural side (Flock and Duvall, 1965; Sobkowicz et al., 1995). The stereocilia and kinocilium of each HC are collectively termed the hair bundle (Figure 1). Adjacent stereocilia are connected by several types of connecting protein including tip links, horizontal top connectors, shaft connectors, and ankle links (Goodyear et al., 2005). Similarly, the kinocilium and adjacent stereocilia are connected by kinocilial links, while in some HCs, the kinocilia are physically separated from stereocilia (Avan et al., 2019). This cilium seems to exhibit a traditional 9 + 2 axoneme pattern in most cases, hence its name (Sobkowicz et al., 1995). However, although it has outer dynein arms and radial spokes, it does not have inner dynein arms (Figure 2; Kikuchi et al., 1989). Therefore, the outer dynein arms allow the kinocilia retaining some motor function to passively swing with the rhythmic vibration rather than through autonomous movement that requires inner dynein arms (Spoon and Grant, 2013).

Mammalian kinocilia mediate HC morphogenesis and PCP, and the latter dictates the proper arrangement of stereocilia that is required for hearing. In mouse cochlear HCs, kinocilium development is complete around embryonic day 15 (E15), after which time they move to the non-neural side of the cell with the basal body. Meanwhile, nearby stereocilia gradually grow to form the three rows of stair-like and V-shaped stereocilia of different heights around E17, together forming the hair bundle (Williams et al., 2017). Kinocilium develops before stereocilia, finally leading the hair bundle facing toward the non-neural side. Accordingly, the HCs also acquire PCP in readiness for hearing and receiving external stimuli. In this way, the kinocilia play vital roles in the maturation of HCs.

The MET apparatuses are located at the top of stereocilia. The hair bundle tilts toward the longer stereocilia when receiving the sound stimulus, and the tip links are stretched, leading to the opening of MET channels and the subsequent depolarization of HCs. Therefore, the stereocilia completely determine the MET activity of mature HCs, so the kinocilium, which dictates the proper arrangement of stereocilia that is required for hearing, must form correctly during the initial stages of HC differentiation. Some typical ciliopathies including Bardet-Biedl syndrome (BBS), Alstrom syndrome (ALMS), Usher syndrome (US) are characterized by hearing dysfunction (Cosgrove and Zallocchi, 2014; Tsang et al., 2018; Hearn, 2019). Mutations in some ciliary genes encoding important intraflagellar transport (IFT) proteins such as *Ift88* can also cause hearing defects. *Ift88* conditional knockout mice exhibit shortened cochlear ducts with multiple extra rows of HCs at the apex, severe hair bundle polarity defects, and premature differentiation of HCs (Moon et al., 2020). Other studies have shown that the phenotypes of these knockout mice all include kinocilium loss, disorderly arrangement of stereocilia of different lengths, short and collapsed structural defects, and mislocation of centrosomes (Tarchini and Lu, 2019). Furthermore, some genes such as *Alms1* encoding proteins associated with centrosomes and ciliogenesis also show abnormal phenotypes in knockout mice, especially the mass loss of OHCs (Jagger et al., 2011). These data indicate that kinocilia play key roles in the correct orientation of stereocilia and consequently the normal function of HCs. Moreover, some ciliopathy related genes encoding connecting proteins can cause hearing dysfunction. Mutation of *Dcdc2a*, which is related to the autosomal recessive deafness-66 and encodes a protein located in the kinocilium, shows deficiency in the regulation of kinocilial ciliogenesis and length, and abnormal cohesion of the kinocilial microtubule core (Grati et al., 2015). Depletion of Usher syndrome 1 (Ush1) proteins, such as CDH23 and PCDH15, which are responsible for tip links and kinocilial links, significantly shortens stereocilia (Cosgrove and Zallocchi, 2014; Richardson and Petit, 2019).

## Degeneration of the Kinocilia

Although kinocilia can be observed in newborn mouse cochlea HCs, they gradually degenerate in HCs from the bottom to the top of the cochlea after mice gain hearing at about postnatal day 8 (P8) and completely disappear at about P12, but the basal body still remains in mature HCs (Leibovici et al., 2005). In contrast, the kinocilia of HCs in the vestibular system persist throughout an animal’s life (Guinan, 2012). The physiological significance of this cochlear degeneration is still not fully understood, but we can gain insights through comparison of cochlear kinocilia with those in the vestibular system.

Kinocilia on the surfaces of the two types of HCs in the vestibular system are anchored in “otolith,” a kind of biomineralized aggregate of calcium and protein (Day and Fitzpatrick, 2005; Ramosdemiguel et al., 2020). When the head inclines or the body accelerates, the otolith shifts due to the effects of gravity, thereby moving kinocilia to one side through kinociliary links (Day and Fitzpatrick, 2005). Coupled with various connections between different parts of stereocilia, the whole hair bundle then leans toward the kinocilium’s bending direction. At that time, as MET channels open, a large amount of K^+^ flows into the hair cells, depolarizing the cell membrane and finally processing the signal of the head position. When the vestibular stimulus disappears, the stereocilia pull the kinocilium in the opposite direction, restoring the cell membrane to its resting potential (Jacobo and Hudspeth, 2014). Surprisingly, although kinocilia are not present in the HCs of the mature cochlea, the stereocilia bundle, after being mechanically stimulated, still oscillates toward the original position of the kinocilium, consistent with the behavior of HCs in the vestibular system (Fettiplace, 2017). Similar to the kinocilium and otolith, the tip of the longest stereocilium in the cochlear HC is anchored to the tectorial membrane above. Structurally, it appears that these stereocilia are substitutes for the vestibular kinocilium. So, do the longest stereocilium and tectorial membrane also have a similar pull-in pattern?

The role of the tectorial membrane in the cochlea helps us to understand this pattern. This membrane links the longest stereocilium of each OHC via otogelin, otogelin-like, and stereocilin proteins (Avan et al., 2019). When sound waves transmit from the perilymph to endolymph, they pass through the basilar membrane as traveling waves, converting vibration to the tectorial membrane via periodic compression at the top of the HC protein network. The relative displacement of both leads to radial fluid flow in a narrow space, a shearing motion, which results in stereocilia movement in the horizontal direction and finally causing stereocilia to tilt (Guinan, 2012). The tectorial membrane acts as a calcium reservoir storing a large amount of Ca^2+^, and HCs can rapidly process signals by directly utilizing the Ca^2+^ released by it instead of relying on endolymph when MET channels open, which may also be the reason why the endolymphatic fluid and the intracellular Ca^2+^ ionic environment are almost the same (Strimbu et al., 2019).

Presumably, kinocilia are not needed for auditory signal processing in the cochlea, since the longest stereocilia play a very similar role. As mentioned above, the cochlea is unique to mammals, and its internal mechanical receptors have correspondingly evolved in structure and function. Primitive vertebrates such as fish only have an inner ear, which is mainly used for balance. Moreover, although they have a complete vestibular system, auditory functions must be taken into account (Whitfield, 2020). Amphibians such as frogs have evolved a middle ear with an eardrum (Mason et al., 2015). In most reptiles, the ear develops further with an internal eardrum, giving rise to a prototypic external auditory canal (Schwab et al., 2020). While the ears of birds and mammals differ greatly, they still have highly developed outer, middle, and inner ears. The cochlea of mammals provides a single organ responsible for hearing that can cooperate with the other sensory functions of the ear. The longest stereocilia and related structures have a very close interrelationship, so the complete degradation of cochlear HC kinocilia will not have a profound physiological effect. This process therefore is probably best regarded a result of evolution.

## Concluding Remarks

Mammalian sensory systems are vital for the interactions between organisms and their environment. Among them, the auditory system is mediated through ion channels and receptors present on the actin filament-based microvilli called stereocilia, in line with the tactile and taste systems. However, unlike the specialized primary cilia present in visual and olfactory systems, the kinocilia are not involved in signal transduction, but play vital roles in mediating precise directional arrangement of stereocilia and the unique distribution of HCs in the cochlea, both of which are crucial for auditory integrity.

Kinocilia have their own unique characteristics that defy their classification into simply “motile” or “primary,” which represents one of the higher evolutionary characteristics of mammals. Unfortunately, evolution can be a double-edged sword. Unlike in some species (such as birds and amphibians) with the capacity for spontaneous regeneration, mammalian cochlear HCs lack the ability to actively regenerate in adults. The irreversible reduction in the number of these sensory cells in some congenital or hereditary genetic diseases or acquired through aging or disease define neurodegenerative pathology. Although perhaps a product of higher evolution of mammals, the physiological significance of kinocilia degeneration is still incompletely understood and further research is necessary to understand the relationship–if any–between kinocilium degeneration and neurodegenerative diseases such as congenital sensorineural hearing loss.

Transplanting sensory cells artificially induced *in vitro* might be one way to restore sensation, and attempts are now underway to generate and then transplant these “simulated cells” to save the loss of HCs. There has been promising progress in using embryonic stem cells and hiPSCs to produce 3D organoids (Liu et al., 2016; Koehler et al., 2017; Jeong et al., 2018). Besides, although various types of cells can be obtained *in vitro* using these tools, their structure, function, and physiological indicators still do not completely replicate *in vivo* conditions. Therefore, artificially generating sensory cells with mature structures and physiological functions for translational use remains an ongoing area of research. With respect to other sensory cells like olfactory sensory neurons, AAV adenoviral -meditated ciliary restoration have shown promise in proof-of-principle preclinical studies (Green et al., 2018; Uytingco et al., 2019). Thus, together with gene editing, *in vitro* gene therapy and stem cell transplantation could become promising therapeutic approaches for overcoming sensorineural loss in the long term, provided that common barriers such as efficacy, safety, and immunorejection are overcome. However, it is still unclear whether kinocilia are present in these organoids. It is also interesting to explore whether mammalian HC regeneration is related to or even regulated by kinocilia. Thus, although kinocilia are promising therapeutic targets for genetic and acquired diseases, further studies are warranted to develop the treatment strategies.

## Author Contributions

DW wrote the manuscript and drew the figures. JZ conceived the study and revised the manuscript. Both authors read and approved the final version of the manuscript.

## Conflict of Interest

The authors declare that the research was conducted in the absence of any commercial or financial relationships that could be construed as a potential conflict of interest.

## Publisher’s Note

All claims expressed in this article are solely those of the authors and do not necessarily represent those of their affiliated organizations, or those of the publisher, the editors and the reviewers. Any product that may be evaluated in this article, or claim that may be made by its manufacturer, is not guaranteed or endorsed by the publisher.
